# Effects of Extruded Linseed and Soybean Dietary Supplementation on Lactation Performance, First-Service Conception Rate, and Mastitis Incidence in Holstein Dairy Cows

**DOI:** 10.3390/ani10030436

**Published:** 2020-03-05

**Authors:** Ahmed Dawod, Hamada Ahmed, Reham Abou-Elkhair, Hamed T. Elbaz, Ayman E. Taha, Ayman A. Swelum, Ibrahim A. Alhidary, Islam M. Saadeldin, Muath Q. Al-Ghadi, Hani A. Ba-Awadh, Elsayed O. S. Hussein, Adham A. Al-Sagheer

**Affiliations:** 1 Husbandry and Animal Wealth Development Department, Faculty of Veterinary Medicine, University of Sadat City, Menoufia 32897, Egypt; adawod@vet.usc.edu.eg; 2 Nutrition and Vet., Clinical Nutrition Department, Faculty of Veterinary Medicine, Damanhour University, Damanhour 22511, Egypt; hamada_nutrition@vetmed.dmu.edu.eg; 3 Nutrition and Clinical Nutrition Department, Faculty of Veterinary Medicine, University of Sadat City, Menoufia 32897, Egypt; reham.abouelkhair@vet.usc.edu.eg; 4 Theriogenology Department, Faculty of Veterinary Medicine, University of Sadat City, Menofia 32897, Egypt; hamed.elbaz@vet.usc.edu.eg; 5 Department of Animal Husbandry and Animal Wealth Development, Faculty of Veterinary Medicine, Alexandria University, Behira, Rasheed, Edfina 22758, Egypt; ayman.taha@alexu.edu.eg; 6 Department of Animal Production, College of Food and Agriculture Sciences, King Saud University, P.O. Box 2460, Riyadh 11451, Saudi Arabia; ialhidary@ksu.edu.sa (I.A.A.); isaadeldin@ksu.edu.sa (I.M.S.); vet.hani77@gmail.com (H.A.B.-A.); shessin@ksu.edu.sa (E.O.S.H.); 7 Department of Theriogenology, Faculty of Veterinary Medicine, Zagazig University, Sharkia 44519, Egypt; 8 Department of physiology, Faculty of Veterinary Medicine, Zagazig University, Sharkia 44519, Egypt; 9 Department of Zoology, College of Science, King Saud University, Riyadh 11461, Saudi Arabia; malghadi@ksu.edu.sa; 10 Animal Production Department, Faculty of Agriculture, Zagazig University, Zagazig 44511, Egypt

**Keywords:** dairy cows, extruded linseed and Soybean, milk yield, milk fatty acids, first-service conception rate

## Abstract

**Simple Summary:**

Natural and processed fat supplements directly affect milk yield and composition in cows. Oilseed extrusion is a heat-treatment process used for seeds fed to ruminants to prevent rumen biohydrogenation of unsaturated fatty acids. This process increases postruminal fat absorption when compared with the effects of feeding whole oilseeds. Hence, we investigated the effects of feeding a mixture of extruded linseed and soybean on lactation performance, first service conception rate, and mastitis incidence in Holstein dairy cows. We found that supplementing dairy cow feed with a mixture of extruded linseed and soybean at a rate of 100 g/kg feed increased milk yield and both monounsaturated and polyunsaturated fatty acid content. However, incidences of clinical mastitis and first-service conception rates were not affected by extruded linseed and soybean supplementation.

**Abstract:**

This study quantifies the effects of extruded linseed and soybean (ELS) dietary supplementation on milk yield, composition, and fatty acid profiles, as well as first-service conception rate in Holstein dairy cows. Seventy-eight open Holstein dairy cows were divided into two groups: (1) a control, which received a basal diet; and (2) a test group, which received a basal diet supplemented with the ELS (650 g/kg of extruded linseed and 150 g/kg of extruded soybean) at a rate of 100 g/kg. In the ELS group, milk yield per day and solid not fat (SNF) yield increased by 3.26% and 0.88%, respectively, in relation to the control. Percentage milk fat decreased significantly by 1.4% in the ELS group when compared with the control. The ELS supplement resulted in a decrease in saturated fatty acids (SFAs) and an increase in monounsaturated (MUFAs) and polyunsaturated fatty acids (PUFAs) in milk. In conclusion, the supplementation of dairy cow feed with 100 g/kg of ELS increases milk yield and milk unsaturated fatty acids (especially MUFAs and PUFAs). ELS supplementation also causes a decrease in percentage fat and SFA levels but does not affect the first-service conception rate or the incidence rate of mastitis.

## 1. Introduction

The onset of lactation is a critical period for high-production dairy cows, when many physiological and nutritional changes tend to occur. A negative energy balance is considered to be a significant hazard facing dairy cows during this period. Therefore, increasing diet energy content may limit the length and severity of this negative energy balance while maintaining milk yield in high-lactating dairy cows. Feeding high-yielding cows with rumen-bypass fats increases the energy content of the diet without the need to increase the starch content or decrease the fiber intake [1]. This avoids disruption of rumen functions [1]. Diet supplementation with fat affects milk yield [2], milk fat [3], and days in milk (DIM) [4]. These effects may depend upon the nature of the fat supplement, how it is processed, and the quantity of fat added to the diet [5].

Linseed is rich in unsaturated fatty acids, as its oil contains approximately 55% α-linolenic acid (ALA) [6]. In ruminant nutrition, when supplementing with unprotected fats, ruminal losses of unsaturated fatty acids can be quantified. These fatty acids undergo a rumen biohydrogenation process before intestinal absorption. This is because the oily nature of these fatty acids interfere with the ruminal microorganism ecosystem [7]. In this respect, Jenkins [8] and Palmquist [9] summarized the ways in which the oily nature of fats may affect microorganisms in the rumen. Initially, fats may be adsorbed onto the bacterial membrane surfaces, consequently lowering microbial activity. This reduces the supply of minerals, such as calcium and magnesium, required for the fermentation activity in the rumen, by the formation of calcium and magnesium soap. In addition, this added fat-antimicrobial effect may be linked to the potential toxicity of various polyunsaturated fatty acids (PUFAs), particularly in cellulolytic bacteria. Indeed, adding polyunsaturated fatty acids to the ruminant diet leads to the formation of many intermediate products created during rumen biohydrogenation. These products include trans-fatty acids and conjugated linoleic acid (CLA) precursors, which may decrease the milk fat percentage [10]. Therefore, the quantity of absorbed PUFAs from the small intestine depends primarily on the degree that PUFAs are protected against ruminal biohydrogenation [11].

Oilseed extrusion is a heat-treatment process for seeds used in ruminant feed. The heat treatment aims to protect unsaturated fatty acids from ruminal biohydrogenation and to safeguard the rumen environment from the adverse effects of fats [12]. Heat treatment of oilseeds denature the proteins, which surround lipid molecules, and consequently reduce ruminal biohydrogenation of fats (and associated unsaturated fatty acids) by rumen microorganisms [11]. Another theory relating to the protection of PUFAs against ruminal biohydrogenation postulates that oilseed extrusion raises the oil release level in rumen fluid [13], and thus leads to greater postruminal fat absorption in comparison to untreated oilseed [14].

Despite there being numerous studies on the effects of extruded linseed [11,12,15,16,17] and extruded soybean [18,19,20] on ruminant feeding, three main questions persist. Firstly, most research findings in this area are contradictory. Secondly, little information is available in relation to the impact of extracted oilseed supplementation on mastitis incidence and reproductive performance. Thirdly, knowledge is limited in relation to the effects of feeding a mixture of extruded linseed and soybean (ELS) on lactation performance, fatty acid profiles, mastitis incidence, and reproductive performance.

Many studies on dairy cows have verified that PUFAs from plant sources have favorable activities as modulators of the immune and inflammatory responses [21,22,23]. Importantly, a negative correlation exists between immune traits and mastitis prevalence [24]. Therefore, based on the previously reported immunomodulatory effects of both extruded oilseeds [25,26], we hypothesize that the use of ELS in dairy cow diets could improve energy density as well as milk fatty acid profiles, while limiting the harmful effects of PUFAs on the rumen environment and its functions. Thus, we quantified the impact of feeding ELS on dairy cow milk yield and composition (including fatty acid profiles), feed efficiency, first-service conception rate, and mastitis incidence.

## 2. Materials and Methods

### 2.1. Animals and Experimental Design

This study was conducted on a private dairy farm located in Damietta province, Egypt. The dairy herd consisted of 1238 Holstein cows with an average milk yield of less than 8000 L per season, a parity number of 2.18 (range 1–9 parity), and a peak milk yield of 38 L. Seventy-eight nonpregnant multiparous Holstein dairy cows were selected from the dairy herd according to their average daily milk yield in the previous 15 days (pretreatment), days in milk (DIM), parity, and reproductive potential. All animal protocols followed the experimentation guidelines of the Internal Commission for Environmental and Ethics of Alexandria University (approval number: ALEXU-0089-2019).

The selected dairy cows were randomly divided into two groups, 39 animals in each. The first served as a control, while the second was assigned as the supplemented group. The control group was fed a lactation ration according to the NRC recommendations [27] for 38 days (Table 1). The second group was fed the same lactation diet supplemented with a Mix-Promega3 dietary supplement (Kemit Co., Cairo, Egypt) at 100 g/kg of dry matter (DM) for 38 consecutive days (Table 1). Mix-Promega3 is a dietary supplement that contains 650 g/kg extruded linseed, 150 g/kg extruded soybean, and 200 g/kg rice bran (details given in Table 2 and Table 3). This supplement is produced by Kemit Co. for Advanced Nutrition (patent no. 3904 at 204,8128). The supplement is composed of seeds which are ground, then mixed and extruded (with extruders using water steam at temperatures greater than 100 °C for 10–30 min). Nutritive values of the tested rations were calculated, including net energy (NE) (i.e., Unité Fourragère Lait (UFL): one UFL = 1700 Kcal of NE) and protein (digested in the small intestine when nitrogen (PDIN) or energy (PDIE) were calculated according to INRA (2010)). The requirements of UFL and PDI for maintenance and milk production were calculated from the body weight (BW), milk yield, and milk protein percentage. The UFL and PDI supplies were calculated using the total mixed ration (TMR) intake and its respective concentrations in UFL and PDI (INRA, 2010). Energy and protein balance were calculated by subtracting energy or protein requirement from their intake.

Before the study began, the average daily milk yields per cow were 30.6 ± 0.1 and 30.4 ± 0.1 kg for the control and the treated groups, respectively. The average DIM was 80.3 ± 6 and 82.4 ± 5 d and the parities were 3.2 ± 0.01 and 3.1 ± 0.01 for the control and the treated groups, respectively. All test subjects from both groups were acclimatized by feeding with the control diet for 15 days before the beginning of experimental treatments. Each group was placed in separate free-stall barns with a 20% roofed area, open sides, and sandy floors. All test subjects were milked three times daily in a herringbone milking parlor equipped with automatic take-off systems and automated recording systems (ALPRO). Milking and machine management practices were conducted according to the standard management system used for Holstein dairy herds.

### 2.2. Ration Analysis

The TMR was offered to each cow group four times per day, and the remaining uneaten rations were weighed at 7 AM the following day. Samples were taken from each ration biweekly and dried for 48 h at 60 °C. By the end of the study, aliquots were taken from each sample for further proximate analyses. Samples were ground and their chemical compositions analyzed according to procedures described by the AOAC [28]. Crude protein (N × 6.25) content was analyzed using the Kjeldahl method (procedure 928.08). Crude fat (procedure 948.15) content was estimated using petroleum ether in a Soxtec System. Acid detergent fiber (ADF) and neutral detergent fiber (NDF) were estimated according to the procedure of Van Soest et al. [29], adapted for the Fiber Analyzer. All chemical analyses were completed using dry matter (DM).

### 2.3. Lactation Performance Data

One dairy cow was removed from the supplemented group during the study due to illness (a severe form of lameness). DIM and parity data were obtained from an on-farm recording system (dairy-Comb). Daily milk yield for each cow was obtained directly from the milking parlor recording system (ALPRO) once per week.

### 2.4. Milk Composition and Fatty Acids Profile

All cows were milked three times per day with eight-hour intervals. Samples of milk were collected biweekly from three consecutive milking events and then pooled for each cow. Bronopol tablets (D&F Control System, San Ramon, CA, USA) were added to each sample as a preservative and then the samples were stored at 4 °C. The samples were analyzed for solid not fat (SNF), protein concentrations, and fat concentrations using the Milko-Scan S50 analyzer (Foss Tecator, Hillerød, Denmark).

Fatty acid composition, in terms of unsaturated fatty acids (USFAs), saturated fatty acids (SFAs), polyunsaturated fatty acids (PUFAs), monounsaturated fatty acids (MUFAs), α-linolenic acid, and conjugated linoleic acid were analyzed using capillary gas chromatography according to the method described in the guidelines of AOAC [28] (procedure 996.01). The analysis was performed in the Faculty of Agriculture, Cairo University, Giza, Egypt. Milk samples were centrifuged (2000× *g*) to extract the fat cake. Lipid extraction was then completed following the method of Hara and Radin [30] by adding 18 mL of isopropanol plus hexane (2:3, *v*/*v*) to each gram of fat cake. The solution was mixed and then 12 mL of 6.7% NaSO_4_ was added. The hexane layer was transferred to a tube containing one gram of NaSO_4_. Once 30 min had elapsed, the hexane layer was extracted and stored at −20 °C until further analysis (methylation).

Fatty acid methylation was conducted according to a method outlined by Ostrowska et al. [31]. Thirty milligrams of the extracted fat from each sample was inserted into 15 mL test tubes fitted with Teflon-lined screw caps. Then, 1.5 mL of 0.5 N NaOH dissolved in methyl alcohol was added. The tubes were flushed with nitrogen, closed, and heated at 100 °C. They were then shaken for 5 min and cooled to room temperature. One milliliter of an internal standard (2 mg heptadecanoic acid, C17:0/mL in hexane) plus 2 mL of boron trifluoride (14%) in methanol were added and the solutions were heated with occasional shaking at 100 °C for 5 min. Ten milliliters of deionized water was then added to each solution. Each solution was transferred to 40 mL centrifuge tubes and 5 mL of hexane was added (to extract fatty acid methyl esters). Afterwards, the solutions were centrifuged (2000× *g*) at 10 °C for 20 min. The hexane layers were dried using sodium sulfate, collected in vials and analyzed using gas chromatography (Hewlett Packard GC system HP6890 A, Hewlett Packard, Avondale, PA, USA). The chromatography machine contained a 100 m × 0.25 mm fused silica capillary column (SP2560, Supelco Inc, Bellefonte, PA, USA). The injector and detector temperatures were 240 °C. During each analysis run, the column temperature was maintained at 70 °C for the first 4 min. It then increased by 13 °C per minute until it reached 175 °C. It was maintained at 175 °C for the next 27 min. The temperature then increased by 4 °C per minute until it reached 215 °C. It was maintained at this temperature for the last 31 min of the run. Pure methyl ester standards (GLC-569, NuChek Prep, Elysian, MN, USA) were used to identify and quantify fatty acid peaks. In order to correct the loss of short-chain fatty acids during the preparation of the sample, butter oil reference standards (CRM 164; Commission of European Communities, Community Bureau of Reference, Brussels, Belgium) were used as an external standard.

### 2.5. First-Service Conception Rate

Artificial insemination of each dairy cow was completed by the herd veterinarian following the appearance of true physical estrus signs. Pregnancies were diagnosed using ultrasonography 28–36 days following the last insemination. The first insemination conception rate was calculated for control and treated groups according to the following formula:

$$\mathrm{Conception}\;\mathrm{at}\;\mathrm{first}\;\mathrm{service}\;\left(\%\right)=\frac{\mathrm{First}\;\mathrm{service}\;\mathrm{conception}}{\mathrm{Total}\;\mathrm{first}\;\mathrm{services}}\times 100$$

### 2.6. Clinical Mastitis

Clinical mastitis cases were suspected when abnormal milk was found during foremilk stripping by milking personnel and confirmed by a veterinarian. The California Mastitis Test was used to confirm diagnoses (where any degrees of reaction were considered positive). Dairy cows with clinical mastitis were treated after each milking session with an intramammary injection containing an approved antibiotic formula (flumethasone, neomycin, and spiramycin) for three days.

### 2.7. Statistical Analysis

The parametric data were analyzed with repeated measures using the PROC MIXED method in the SAS software [32]. All continuous parameters (daily milk yield, milk solids) were covariables and were calculated from 15 days before the treatments began. Milk yields and milk fatty acid profiles were analyzed using General linear models (GLMs), and the results were represented as least square means and SEM. The GLM model structures were as follows:

Y_ijkl_ = μ + T_i_ + P_j_ + C (T × P)_ijk_ + DIM_ijkl_ + E_ijklm_

where μ = overall mean, T_i_ = treatment effect, i = 1 or 2, P_j_ = parity, j = 2 or greater, C (T × P)_ijk_ = cow k nested in treatment i and in parity j; DIM_ijkl_ = days in milk; and E_ijklm_ = a random residual.

Once the main treatment effect was found to be significant, a pairwise t-test (the ‘pdiff’ option of PROC MIXED, SAS software) was performed to separate means. The clinical mastitis incidences in different groups were analyzed using the chi-square test. Explanatory variables with two levels, e.g., first-service conception rate (pregnancy and nonpregnancy) and mastitis incidence (infected and noninfected), were analyzed using a categorical method (chi-square analyses).

## 3. Results

### 3.1. Milk Production and Feed Efficiency

There were significant differences in dairy cow performance in response to ration supplementation with ELS (Table 4). Dairy cows fed rations supplemented with ELS showed significant increases in average daily milk yields (3.26%; *p* = 0.02) and percentage SNF (0.88%; *p* = 0.05) compared with cows in the control group (Table 4). In contrast, a significant (*p* = 0.01) reduction in milk fat percentages (1.40%) was observed in the ELS group compared to the control (Table 4). No significant differences were detected in percentage milk proteins between the experimental diets (Table 4). However, a significantly (*p* = 0.04) higher protein yield per day was observed in the ELS group compared to the control (Table 4). The total fat and SNF yield per day were also significantly higher in the ELS group compared to the control (Table 4). There were no differences between groups in terms of feed intake or feed efficiency (Table 4). Both the experimental diets supplied similar amounts of energy (UFL) but significantly (*p* = 0.047) different quantities of PDIE (Table 4). Based on calculations incorporating milk yield and feed intake, the control diet did not supply the full energy requirements of the test subjects. The supplied amounts of PDIE were significantly higher in the ELS group compared to the control. However, energy and protein balance significantly (*p* < 0.001) increased in the cows fed the ELS diet compared with the control diet.

### 3.2. Milk Fatty acid Profile

The results of milk fatty acids (FAs) analyses are presented in Table 5. The milk fat of cows fed with ELS had lower C16:0 and C18:0 concentrations than those in the control. In contrast, higher milk fat content of C18:1 trans-11, C18:2 cis-9, cis-12, and C18:2 cis-12, trans10 were detected in the ELS group than in the control. We found that α-linolenic acid (C18:3 n-3) concentrations increased significantly in the ELS group compared with the control. Also, total UFAs increased significantly from 241.10 g/kg in the control group to 345.2 g/kg in the ELS group. The ELS supplement resulted in a decrease in SFAs and an increase in MUFAs and PUFAs compared with the control. Furthermore, the conjugated linoleic acid (C18:2 cis-9, trans11) concentrations were significantly higher in the ELS group than in the control. The milk fat of cows fed with ELS had a higher LA/ALA ratio compared with cows in the control group.

### 3.3. First-Service Conception Rate and Mastitis Incidence

As shown in Table 6, the results of the first service conception rates in dairy cows showed no significant differences (chi-square χ^2^ = 0.051, *p* = 0.822) between means during the first 80–110 DIM. Additionally, the incidence rate of clinical mastitis was not significantly affected (chi-square χ^2^ = 0.402, *p* = 0.53) by ELS supplementation compared with the control (Table 7).

## 4. Discussion

Milk quality is one of the most important critical issues affecting the dairy industry. The present study aimed to improve milk quality and dairy cows’ productive and reproductive performances by supplementing their diets with ELS. Cows that were fed this supplemented diet for 38 consecutive days were found to increase their milk production by 3.26% relative to the control group. This result is consistent with previous studies [11,17] but contrasts with several others [15,16,33,34] that researched the use of extruded linseed (EL) in cow diets. Our finding in relation to the increased milk yields in the ELS group may be attributed to the presence of extruded soybean in our supplemented diet (regardless of the small proportion included in the mixture). Giallongo et al. [18] suggested that increases in milk yield due to dietary extruded soybean supplementation may be related to an increase in dry matter, crude protein, and fiber intake. In the current investigation, PDIE intake as well as energy and protein balance were increased in the ELS group, which likely clarifies the variances in milk production between the experimental diets.

In the present study, supplementation with ELS decreased the percentage of milk fat. This is consistent with some previous studies that found that diets supplemented with extruded linseed resulted in a decrease in the percentage of milk fat [17,35]. However, this decrease was not recorded in other studies that supplemented cow diets with whole [33,36] or extruded linseed [17,37]. We found that supplementing cow diets with ELS did not affect milk protein concentrations or yields. This result is consistent with previous studies [33,36,37].

Our observation that milk fat content of C16 and C18 decreased with ELS feeding is contradictory with the results of other studies [38,39]. This response might be related to higher milk fat contents of C18:2 trans-10, cis-12 [40]. Several studies have found that feeding diets containing EL caused a reduction in SFA concentrations and an increase in MUFA concentrations [15,17,35,37], similar to the findings in the present study. We found that the concentrations of PUFAs, ALA, and CLA in dairy cows’ milk increased following diet supplementation with ELS. These results are consistent with previous studies [11,15,37]. Neveu et al. [15] found that EL supplementation improves milk fat USFAs, α-linolenic acid, and conjugated linoleic acid in the milk of dairy cows.

In this study, DM intake did not differ between the ELS and the control groups, and this is consistent with previous studies [41,42,43]. Previous studies also reported no change in the intakes of DM and NEL when the concentration of total fat was less than 6% of DM. In contrast, Martin et al. [44] found that supplementation with linseed oil at a rate of 5.7% DM caused a significant decrease in dry matter intake (DMI). However, they also found that supplementation of the dairy ration with either crude or extruded linseed did not affect DMI. Similar outcomes were reported by Lerch et al. [33].

Once dairy cows have calved, successful conception following the first service is essential in order to maximize reproductive efficiency [45]. Lower conception rates after the first service may result in an increase in repeated insemination procedures, days open, reproductive medication, culling, and replacement heifers [46]. In this study, the first service conception rate ranged from 40.91% in Holstein dairy cows fed the control diet, and 44.44% in cows fed the ELS diet. This range is within the range of 26.7% to 50.7% reported by previous studies [47,48]. Our finding that the first-service conception rate of dairy cows did not alter depending on the experimental diets is consistent with previous studies that reported conception and pregnancy rates to be unchanged when diets were supplemented with linseed, saturated FA, or n-6 FA [41,49]. Similarly, in another study, there was no significant difference in conception rates at first service when lactating cows were fed a diet supplemented with rolled flaxseed versus a control [50]. In contrast, some studies have found that feeding extruded linseed to dairy cows was associated with a reduced number of days required before first artificial insemination as well as reduced days to conception, but they did not relate diet to the overall rate of return to service [51,52]. The lack of effects of ELS on first-service conception rates in the present study may be attributable to the observed improvements in milk production in ELS-supplemented cows, which resulted in a negative energy balance. In addition, if, as in our study, a low number of cows were subjected to a first service, this may have contributed to the fact that there was no difference in the conception rate between the ELS-supplemented cows (44.4%) and those in the control (40.91%) [53].

Several studies on dairy cows have confirmed that PUFAs from plant sources have beneficial effects as modulators of the inflammatory and immune responses [21,22,23]. A negative correlation has been found between mastitis incidence and immune traits [24]. It is thought that n-3 fatty acids may influence cell immunity in the mammary gland. In this context, the lymphocyte proliferative reaction was found to decrease in dairy cows subjected to a linseed treatment five days after calving [23]. Mach et al. [54] observed that mammary gland immune function in midlactation cows was positively affected by diet supplementation with unsaturated fatty acids. In this way, mastitis susceptibility may be reduced. Surprisingly, in our study, mastitis incidence in dairy cows was not significantly different between the experimental groups. To our knowledge, there are few studies to date on the effects of extruded oilseed supplementation on the incidence of mastitis in dairy cows. The reasons underlying the absence of a significant ELS effect on mastitis incidence in the current study are unknown. However, as for during the lactation stage, the reasons may be related to the concentration used or the low number of lactating cows included in our study. Thus, we recommended that future studies on this issue incorporate a larger number of lactating cows in different stages of lactation and various ELS concentrations into their experimental designs.

## 5. Conclusions

The inclusion of a mixture of extruded linseed and soybean in dairy cow diets at a rate of 100 g/kg increased milk yield and SNF, but decreased the percentage of milk fat in our study. However, milk fat quality increased due to ELS supplementation in terms of USFAs (and its two components MUFA and PUFA). ELS supplementation increased the proportion and the yield of both ALA and CALA. No significant effects on the first-service conception rate and the incidences of mastitis were observed as a result of ELS supplementation. Thus, from both a production and a health point of view, there are numerous benefits to using ELS as a supplement in dairy cow diets. Further experimental trials on a large scale are recommended to reassess the results, particularly in relation to first-conception rate and the incidence of mastitis.

## Figures and Tables

**Table 1:** Ingredients and nutrient composition (g/kg) of the two experimental diets (control and supplemental).

Ingredients	Control	Supplemental	Nutrient Composition	Control	Supplemental
Corn grain, ground	225	269	Organic matter	913.59	916.19
Soybean meal	49	39	Crude protein	145.9	142.33
Cottonseed	52	36	Crude fat	27.11	37.40
Wheat bran	120	120	NDF	291.91	305.73
			ADF	192.42	189.22
Rice bran	107.5	89.5	Hemicellulose	99.51	116.54
Corn silage	200	200	Cellulose	152.80	151.55
Alfalfa fresh	200	200	Lignin	39.62	37.63
Molasses	5	5	NDICP	24.73	25.71
Salt	4	4	ADICP	16.51	14.22
Calcium salt of fatty acids	0.5	0.5	NFC	398.82	390.88
Dicalcium phosphate	5	5	UFL (per kg)	0.72	0.74
Limestone	25	25	PDIN (g/kg)	81.66	82.09
Sodium bicarbonate	6	6	PDIE (g/kg)	72.54	73.11
Vitamins and minerals	1	1			
Mix-Promega 3	-	100			

Note: NDF, neutral detergent fiber; ADF, acid detergent fiber; NDICP, neutral detergent insoluble protein; ADICP, acid detergent insoluble protein; NFC, nonfiber carbohydrate; UFL, Unité Fourragère Lait; PDIN, protein digested in the small intestine following certain nitrogen calculations; PDIE, protein digested in the small intestine following certain energy calculations (see text).

**Table 2:** Amino acid, mineral, and vitamin concentrations in the extruded linseed and soybean supplement.

Amino Acid	g/kg	Mineral	Amount	Vitamin	mg/kg
Lysine	14.32	Calcium (g/kg)	2.2	Carotene	0.32
Methionine	4.74	Phosphorus (g/kg)	6.5	Vitamin A (IU)	7.10
Cysteine	4.64	Phosphorus Available (g/kg)	1.3	Vitamin E	16.76
Threonine	10.82	Chlorine (g/kg)	0.3	Biotin	0.11
Tryptophan	3.61	Magnesium (g/kg)	3.8	Folic acid	1.16
Isoleucine	12.98	Potassium (g/kg)	15	Niacin	20.34
Leucine	18.95	Sodium (g/kg)	1.6	Pantothenic acid	5.10
Valine	14.52	Sulfur (g/kg)	2.6	Riboflavin	2.36
Histidine	6.80	Cobalt (mg/kg)	0.09	Thiamin	5.39
Arginine	24.72	Copper (mg/kg)	34.44	Pyridoxine	3.23
Glycine	12.88	Iodine (mg/kg)	0.14	Choline (g)	1.88
Serine	14.73	Iron (mg/kg)	162.74		
Phenylalanine	14.01	Manganese (mg/kg)	46.28		
Tyrosine	7.62	Selenium (mg/kg)	29.42		
Aspartic acid	29.05	Zinc (mg/kg)	32.58		
Glutamic acid	52.12				
Proline	11.85				
Alanine	12.26				

**Table 3:** Nutrient and fatty acid concentrations in the extruded linseed and soybean supplement.

Nutrient	g/kg Dry Matter	Fatty Acid	g/kg Fatty Acid
Crude protein	310.0	Palmitic acid (16:0)	124.15
Crude fat	289.9	Stearic acid (16:1)	56.42
Ash	51.6	Oleic Acid (18:1)	236.73
Crude fiber	119.7	Linoleic acid (18:2)	267.63
ADF	169.2	Linolenic acid (18:3)	288.05
NDF	237.3	CLA	27.01
TDN	108.79	SFA	124.15
NEg (Mcal/kg)	2.60		
NEL (Mcal/kg)	2.45		

Note: TDN, total digestible nutrient; NEg, net energy gain; NEL, net energy lactation; ADF, acid detergent fiber; NDF, neutral detergent fiber; CLA, conjugated linoleic acid; SFA, saturated fatty acid.

**Table 4:** Mean feed efficiency, milk yield, and milk composition following the supplementation of Holstein dairy cow diets with extruded linseed and soybean (ELS) (n = 38) versus a control (n = 39).

Milk Yield and Composition	Control	ELS	SEM ^1^	*p*-Value
Milk yield (kg/d)	30.70	31.70	0.03	0.02 *
Fat yield (g/d)	988.36	1023.16	5.96	0.04 *
Protein yield (g/d)	1021.06	1041.01	8.38	0.04 *
Solid not fat yield (g/d)	2680.28	2796.04	15.27	0.05 *
Milk protein (%)	3.48	3.48	<0.00	0.70
Milk fat (%)	3.58	3.53	<0.00	0.01 **
Solid not fat (%)	9.04	9.12	0.01	0.04 *
Feed intake (kg/d)	25.11	26.20	0.86	0.87
Feed efficiency	1.39	1.41	0.21	0.98
Energy and protein supply				
UFL (per day)	18.08	19.39	0.48	0.058
PDIN (g/day)	2050	2151	53.02	0.182
PDIE (g/day)	1821	1915	32.95	0.047 *
Energy and protein balance ^2^				
Energy balance (UFL)	−0.12	0.75	0.11	<0.001 ***
Protein balance (g PDI)	15.23	54.19	0.19	<0.001 ***

Note: ^1^ SEM shows the standard error of the mean, ^2^ energy or protein requirement minus intake, * *p* ≤ 0.05, ** *p* ≤ 0.01, *** *p* ≤ 0.001.

**Table 5:** Milk fatty acid composition and mean concentrations in control and supplemented (ELS) diets given to Holstein dairy cows.

Fatty Acid (g/100 g)	Control	ELS	SEM ^1^	*p*-Value
C4:0	5.32	4.29	0.42	0.114
C6:0	2.12	1.80	0.21	0.307
C8:0	1.14	1.02	0.10	0.716
C10:0	1.84	1.6	0.15	0.584
C12:0	2.75	2.52	0.18	0.668
C14:0	8.45	7.93	0.44	0.822
C14:1 cis-9	0.71	0.87	0.18	0.758
C15:0	0.49	0.48	0.07	0.924
C16:0	34.6	25.37	0.61	0.001 **
C16:1 cis-9	1.27	1.43	0.16	0.471
C18:0	16.64	12.8	0.67	0.019 *
C18:1 trans11	2.01	3.21	0.27	0.035 *
C18:1 cis-9	17.35	24.32	0.83	0.001 **
C18:2 cis-9, 12	1.62	2.04	0.07	0.005 **
C18:2 cis-9, trans11	0.21	0.60	0.01	<0.001 ***
C18:2 cis-12, trans10	0.18	0.41	0.04	0.007 **
C18:3 n-3	0.32	1.04	0.01	<0.001 **
C18:3 n-6	0.27	0.33	0.02	0.078
C20:0	0.77	0.58	0.10	0.228
C20:1	0.17	0.27	0.04	0.128
Σ SFA	74.12	58.39	0.72	0.005 **
Σ UFA	24.11	34.52	0.22	0.003 **
Σ MUFA	21.51	30.10	0.36	0.007 **
Σ PUFA	2.6	4.42	0.04	<0.001 ***
LA/ALA ratio	5.06	1.96	0.21	0.002 **

Note: ^1^ SEM shows the standard error of the mean. SFA, saturated fatty acid; USFA, unsaturated fatty acids; MUFA, monounsaturated fatty acids; PUFA, polyunsaturated fatty acids. * *p* ≤ 0.05, ** *p* ≤ 0.01, *** *p* ≤ 0.001.

**Table 6:** Conception rate from first service in Holstein dairy cows fed diets supplemented with extruded linseed and soybean (ELS) versus a control. Data analyzed using a chi-square test.

Experimental Group	Number of Cows Subjected to a First Service (N = 40)	Number (Percentage) Pregnant		Number (Percentage) Not Pregnant		Chi-Square χ^2^	*p*-Value
Control	22	9	(40.91)	13	(59.09)	0.051	0.822
ELS	18	8	(44.44)	10	(55.56)		

**Table 7:** Clinical mastitis incidences in Holstein dairy cows fed diets supplemented with extruded linseed and soybean (ELS) compared to a control. Data analyzed using a chi-square test.

Experimental Group	Number of Cows (N = 78)	Mastitis Incidence		Chi-Square χ^2^	*p*-Value
		Frequency	%		
Control	39	6	15.38	0.402	0.530
ELS	38	4	10.53		
